# Effects of Glutamine and Alanine Supplementation on Central Fatigue Markers in Rats Submitted to Resistance Training

**DOI:** 10.3390/nu10020119

**Published:** 2018-01-25

**Authors:** Audrey Yule Coqueiro, Raquel Raizel, Andrea Bonvini, Thaís Hypólito, Allan da Mata Godois, Jéssica Ramos Rocha Pereira, Amanda Beatriz de Oliveira Garcia, Rafael de Souza Bittencourt Lara, Marcelo Macedo Rogero, Julio Tirapegui

**Affiliations:** 1 Department of Food and Experimental Nutrition, Faculty of Pharmaceutical Sciences, University of São Paulo, Avenida Professor Lineu Prestes 580, São Paulo SP 05508-000, Brazil; abonvini@usp.br (A.B.); jessicarrp3@gmail.com (J.R.R.P.); amandabogarcia@gmail.com (A.B.d.O.G.); rafael.souza.b.lara@hotmail.com (R.d.S.B.L.); tirapegu@usp.br (J.T.); 2 Department of Nutrition, Faculty of Public Health, University of São Paulo, Avenida Doutor Arnaldo 715, São Paulo SP 01246-904, Brazil; thaishypolito@gmail.com (T.H.); mmrogero@usp.br (M.M.R.); 3 Faculty of Nutrition, Federal University of Mato Grosso, Avenida Fernando Correa 2367, Cuiabá MT 78060-900, Brazil; allangodois@hotmail.com

**Keywords:** glutamine, alanine, resistance training, fatigue, central fatigue, mental fatigue

## Abstract

Recent evidence suggests that increased brain serotonin synthesis impairs performance in high-intensity intermittent exercise and specific amino acids may modulate this condition, delaying fatigue. This study investigated the effects of glutamine and alanine supplementation on central fatigue markers in rats submitted to resistance training (RT). Wistar rats were distributed in: sedentary (SED), trained (CON), trained and supplemented with alanine (ALA), glutamine and alanine in their free form (G + A), or as dipeptide (DIP). Trained groups underwent a ladder-climbing exercise for eight weeks, with progressive loads. In the last 21 days, supplementations were offered in water with a 4% concentration. Albeit without statistically significance difference, RT decreased liver glycogen, and enhanced the concentrations of plasma glucose, free fatty acids (FFA), hypothalamic serotonin, and ammonia in muscle and the liver. Amino acids affected fatigue parameters depending on the supplementation form. G + A prevented the muscle ammonia increase by RT, whereas ALA and DIP augmented ammonia and glycogen concentrations in muscle. DIP also increased liver ammonia. ALA and G + A reduced plasma FFA, whereas DIP increased this parameter, free tryptophan/total tryptophan ratio, hypothalamic serotonin, and the serotonin/dopamine ratio. The supplementations did not affect physical performance. In conclusion, glutamine and alanine may improve or impair central fatigue markers depending on their supplementation form.

## 1. Introduction

Central fatigue is a multiple-cause phenomenon characterized by low energy availability [1,2,3], ammonia accumulation in blood and tissues [4], and changes in neurotransmitter synthesis, such as the increase in serotonin and the decrease in dopamine, which cause a state of tiredness, sleep, and lethargy during exhaustive exercise [5,6,7,8,9,10,11].

The underlying mechanisms behind the increase in brain serotonin are the plasma increase in its precursor, free (not albumin-bound) tryptophan, and the plasma reduction in the large neutral amino acids, such as branched-chain amino acids (BCAA), which compete with tryptophan to enter the brain. Additionally, during prolonged exercise, the increase in free fatty acid (FFA) levels can displace tryptophan from albumin, increasing free tryptophan and facilitating its brain influx [8,12,13,14,15,16,17].

Although the parameters involving increased brain serotonin are commonly evaluated in endurance exercise, it was recently observed that the decrease in the plasma-free tryptophan/BCAA ratio improved physical performance in high-intensity intermittent exercise, suggesting that serotonin synthesis may be involved in fatigue development in other types of exercise, such as in resistance training (RT) [18,19].

In order to improve exercise performance, some ergogenic aids, such as glutamine and alanine, are used. These amino acids are linked to the reduction in fatigue markers through several mechanisms: (i) both have a significant influence on the anaplerosis of the tricarboxylic acid cycle and gluconeogenesis, which could attenuate lipolysis and the use of BCAA as muscle energetic substrates, maintaining the plasma concentrations of free tryptophan [20,21,22,23]; (ii) glutamine stimulates glycogen synthesis by the activation of glycogen synthase [20]; (iii) through the supply of carbon skeletons for the energy synthesis, both amino acids have a protective effect against increased ammonia synthesis [23]; (iv) glutamine and alanine are non-toxic ammonia transporters, carrying ammonia from muscle to the liver and kidneys to be metabolized and excreted, respectively, preventing this metabolite accumulation [24,25]; and (v) glutamine synthesis is the main mechanism of detoxification when brain ammonia content increases [26], among others.

Glutamine supplementation is popular in sports nutrition [27,28,29], although the efficacy of its administration in the free form is controversial in the literature [30,31,32]. Previous studies in our laboratory showed that supplementation with the dipeptide l-alanyl-l-glutamine was more effective in increasing plasma, muscle and liver glutamine concentrations when compared to free glutamine administration [31,33]. Notwithstanding, a solution containing glutamine and alanine in their free form presented similar effects relative to l-alanyl-l-glutamine in increasing glutamine availability [34]. We recently observed that alanine supplementation alone also increased plasma and tissue glutamine, albeit supplements containing both glutamine and alanine were better at improving immune and oxidative parameters [27,28]. Nevertheless, little is known about the role of alanine, directly or indirectly (by increasing glutamine availability), in fatigue markers and exercise performance.

Overall, this study aimed to evaluate the effects of glutamine and alanine supplementation in their free or conjugated form on central fatigue markers of rats submitted to progressive RT. We hypothesized that these amino acids could maintain plasma free tryptophan content by reducing lipolysis and BCAA use as energy substrates, preventing the increase in brain serotonin synthesis, in addition to reducing ammonia accumulation and glycogen depletion. Finally, we also hypothesized that the improvement of these parameters could delay central fatigue, thereby increasing physical performance.

## 2. Material and Methods

### 2.1. Animals

Forty adult male Wistar rats, aged two months, and weighing approximately 300 g, were used in this study. The animals were provided by the animal house of the University of São Paulo and housed two per cage in a controlled environment at 22 ± 2 °C and relative air humidity of 55 ± 10%, under a 12-h light/12-h dark cycle (lights on 6 pm, lights off 6 am) for a period of eight weeks. The animals were allowed to acclimate to the conditions for one week before the beginning of the experimental protocol.

The rats were divided into five groups: SED (sedentary and without supplementation/*n* = 8), CON (trained and without supplementation/*n* = 8), ALA (trained and supplemented with l-alanine/*n* = 7), G + A (trained and supplemented with l-glutamine and l-alanine in their free form/*n* = 8), and DIP (trained and supplemented with the dipeptide l-alanyl-l-glutamine/*n* = 8).

The animals had free access to water and standard chow composed of 22% of protein/kg (NUVILAB CR1, Nuvital Nutrients, Curitiba, Brazil). Food consumption, fluid intake, and body weight were registered three times per week. All procedures were approved by the Ethics Committee on Animal Use of the University of São Paulo, according to the guidelines of the Brazilian College of Animal Experimentation (protocol: CEUA/FCF/532).

### 2.2. Resistance Training

The RT protocol was adapted from Scheffer et al., [35], originally published by Hornberger and Farrar [36], and consisted of climbing a vertical ladder (1.1 × 0.18 m, 2 cm grid, 80° inclined) with a load affixed to the base of the rat’s tail, using plastic insulation tape, for a duration of eight weeks.

**Figure 1:**
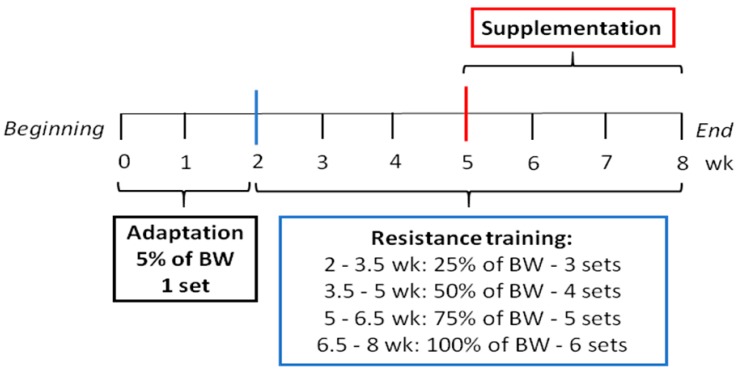
Resistance training protocol and experimental design. Abbreviations: BW: body weight; WK: week.

Each set consisted of eight climbs and a two-minute rest between sets. The adaptation period consisted of one set, with a load equal to 5% of body weight (BW), for two weeks. After this period, RT was conducted with progressive loads (three sets with 25% of BW; four sets with 50% of BW; five sets with 75% of BW; and six sets carrying 100% of BW). Loads and sets were progressively increased every one and a half weeks. The animals were trained during the dark cycle, three times per week, with a duration of approximately 40 min per exercise session and 48 h rest between sessions (Figure 1).

### 2.3. Maximum Carrying Capacity (MCC) Tests

Three MCC tests were performed during the experimental protocol (after the adaptation period, before supplementation and prior to euthanasia). The test consisted of a first climb with 75% of BW, and an addition of 15% of BW on each climb until exhaustion (MCC), with two minutes rest between climbs [37].

### 2.4. Supplementation

Animals were supplemented chronically for 21 days [31,33,38,39], from the 5th week until the day before euthanasia (8th week). Supplements were given in drinking water, diluted to a 4% concentration (4 g dissolved in a final volume of 100 mL of fresh water), provided *ad libitum* and changed three times per week. Supplementation in water was selected in an attempt to reduce the stress of manipulation in oral gavages and to increase the frequency of amino acid intake throughout the day [40].

The amino acid amount was calculated based on a commercial dipeptide concentration (Dipeptiven^®^ a solution containing 20 mg of l-alanyl-l-glutamine dissolved in 100 mL of water, which is equal to 8.21 g of l-alanine and 13.46 g of l-glutamine). Free l-glutamine and free l-alanine were manufactured by Labsynth (Synth, São Paulo, SP, Brazil), and l-alanyl-l-glutamine was manufactured by Fresenius Kabi S.A. (Bad Homburg, HE, Germany). Animals from the SED and CON groups had access to the same amount of fluid, containing only fresh water.

### 2.5. Plasma Parameters

Glycemia was determined in the fourth exercise session of each load: 25, 50, 75, and 100% of BW, before and after training. Samples were collected from the tail vein into tubes containing a sodium fluoride solution and assayed using the enzymatic kit (Labtest, Lagoa Santa, Brazil).

Animals were fasted one hour before training to prevent the influence of food consumption on energy metabolism parameters, such as glycemia and glycogen. One hour after the last exercise session, rats were anesthetized with rodent cocktail—xylazine hydrochloride (20 mg/mL) and ketamine hydrochloride (70 mg/mL)—and then euthanized by decapitation. Blood was centrifuged for plasma separation, and samples were stored at −80 °C for further analyses.

Free fatty acids (FFA) (Cayman Chemical, Ann Arbor, MI, USA) and total and free tryptophan (Abcam Discover More, Cambridge, UK) were measured by the fluorometric method. Glycemia (Labtest, Lagoa Santa, Brazil) and ammonia (Sigma-Aldrich, St. Louis, MO, USA) were assayed by enzymatic kits. Amino acids concentrations were measured by the high-performance liquid chromatography (HPLC) method, from the CBO—Análises Laboratoriais Ltd. Company (Valinhos, Brazil).

### 2.6. Tissue Measurements

Hypothalamus, liver, and tibialis anterior skeletal muscle were surgically excised after euthanasia, weighed, and immediately frozen in liquid N_2_ for subsequent analysis. Serotonin and dopamine were measured in the hypothalamus by an enzymatic kit, according to the manufacturer’s instructions (IBL, Hamburg, Germany). Ammonia was measured in the liver and tibialis muscle using an enzymatic kit (Sigma-Aldrich, St. Louis, MO, USA). Glycogen content in liver and tibialis muscle was measured according to Hassid [41].

### 2.7. Statistical Analysis

Comparisons between groups were carried out by one-way ANOVA with Tukey’s honestly significant differences (HSD) as a post hoc test. Analyses over multiple time points were performed with two-way ANOVA with Tukey’s HSD as a post hoc test. A significant level of *p* < 0.05 was applied for all comparisons. Data were analyzed using GraphPad Prism software, version 6.0 (GraphPad Software, San Diego, CA, USA) and expressed as mean values and standard deviations (SD).

## 3. Results

### 3.1. Food Consumption, Fluid Intake, and Body Weight Gain

There was no difference between groups in terms of food consumption (*p* = 0.082) and baseline body weight (*p* = 0.056). Supplemented animals, especially with G + A, ingested more fluid than the SED and CON groups (*p* < 0.001). As expected, the CON group significantly gained less weight than SED animals (*p* = 0.044) (Table 1).

### 3.2. Plasma Amino Acids

There was no statistically significant difference between groups in terms of plasma concentrations of amino acids, except for tryptophan. Interestingly, animals supplemented with DIP presented the lowest levels of total plasma tryptophan (*p* = 0.022), but the free tryptophan/total tryptophan ratio was the highest in this group (*p* = 0.017), evidencing that a large proportion of the total tryptophan was not bound with albumin (Table 2).

### 3.3. Glycemia, Glycogen, and Free Fatty Acids (FFA)

Glycemia increased in the post-training of all loads (25, 50, 75, and 100% of BW), and there was no statistically significant difference between groups (Table 3).

**Figure 2:**
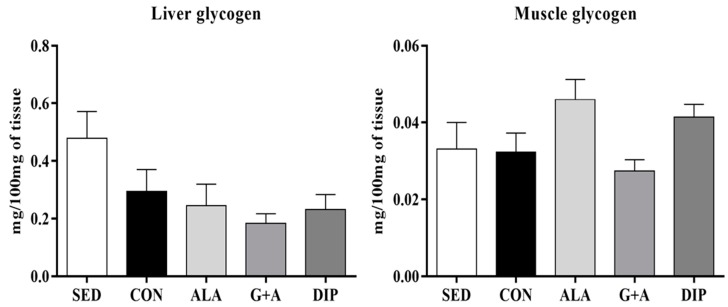
Liver and muscle glycogen of rats submitted to resistance training and supplementation with l-glutamine and l-alanine. SED: sedentary group, CON: trained group, ALA: trained and supplemented with alanine, G + A: trained and supplemented with glutamine and alanine in their free form, and DIP: trained and supplemented with l-alanyl-l-glutamine. Data are presented as mean ± SD.

Albeit, without statistically significant difference, RT reduced liver glycogen content by approximately 52% (SED vs. trained groups), but the amino acids supplementation did not improve this parameter (*p* = 0.086). On the contrary, animals from the ALA and DIP groups presented higher muscle glycogen concentrations, 41% and 28%, respectively, compared with the CON group, although with no statistically significant difference (*p* = 0.051) (Figure 2).

**Figure 3:**
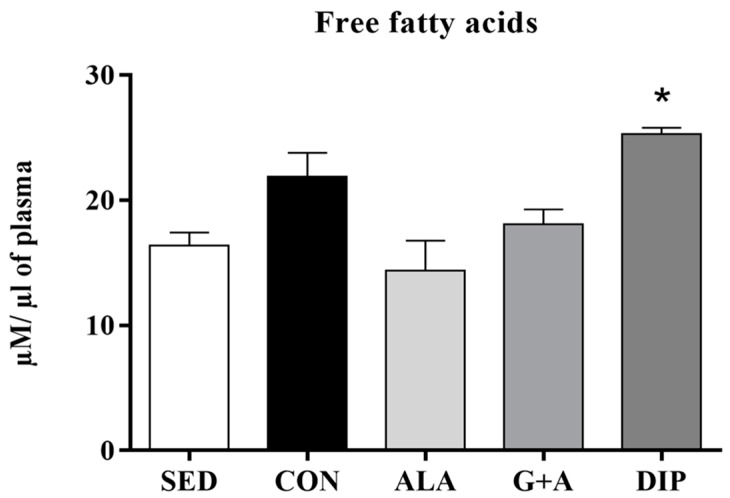
Plasma free fatty acids of rats submitted to resistance training and supplementation with l-glutamine and l-alanine. SED: sedentary group, CON: trained group, ALA: trained and supplemented with alanine, G + A: trained and supplemented with glutamine and alanine in their free form, and DIP: trained and supplemented with l-alanyl-l-glutamine. * *p* < 0.05 vs. SED and ALA. Data are presented as mean ± SD.

RT slightly increased plasma FFA, by 33% (SED vs. CON), while glutamine and alanine supplementation, in their free form (ALA and G + A groups), reduced this parameter by 34% (ALA vs. CON) and 17% (G + A vs. CON). Unexpectedly, the DIP group presented the highest plasma FFA values, 53% higher than SED and 74% higher than ALA (*p* = 0.002) (Figure 3).

### 3.4. Hypothalamic Serotonin, and Dopamine

**Figure 4:**
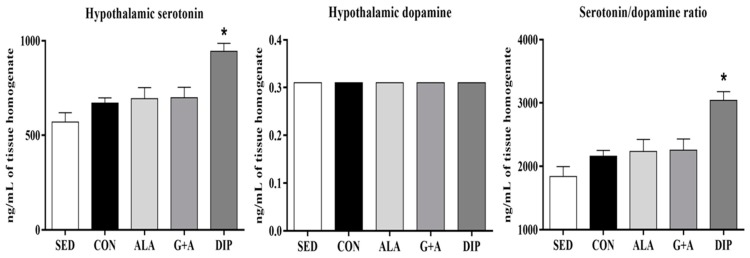
Hypothalamic serotonin, dopamine, and serotonin/dopamine ratio of rats submitted to resistance training and supplementation with l-glutamine and l-alanine. SED: sedentary group, CON: trained group, ALA: trained and supplemented with alanine, G + A: trained and supplemented with glutamine and alanine in their free form, and DIP: trained and supplemented with l-alanyl-l-glutamine. * *p* < 0.05 vs. SED and CON. Data are presented as mean ± SD.

Similarly to the results obtained in plasma FFA, in our study, RT increased hypothalamic serotonin by 17% (SED vs. CON), and DIP supplementation increased serotonin content in the hypothalamus, compared with animals from the SED and CON groups (*p* = 0.001). Concerning the hypothalamic dopamine, there was no difference between groups, demonstrating that neither RT nor supplementation affected this parameter (*p* = 0.865). Thus, the results of serotonin/dopamine ratio were similar to those found in hypothalamic serotonin (*p* = 0.001) (Figure 4).

### 3.5. Plasma, Muscle, and Liver Ammonia

**Figure 5:**
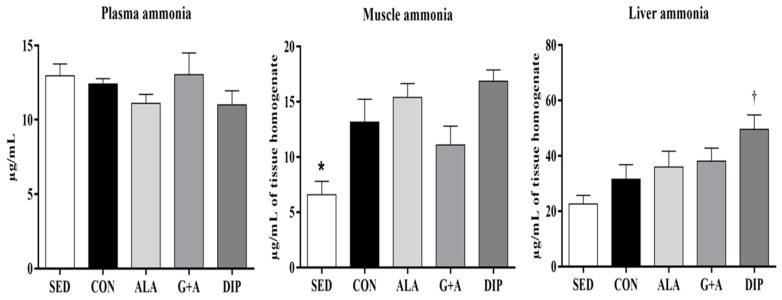
Plasma, muscle, and liver ammonia of rats submitted to resistance training and supplementation with l-glutamine and l-alanine. SED: sedentary group, CON: trained group, ALA: trained and supplemented with alanine, G + A: trained and supplemented with glutamine and alanine in their free form, and DIP: trained and supplemented with l-alanyl-l-glutamine. * *p* < 0.05 vs. CON, ALA, and DIP. † *p* < 0.05 vs. SED. Data are presented as mean ± SD.

Plasma ammonia did not differ between groups (*p* = 0.336). On the other hand, RT increased muscle ammonia in the CON (100%), ALA (133%), and DIP (156%) groups, compared with sedentary animals, while G + A supplementation prevented the effect of RT on increasing muscle ammonia (*p* = 0.001). Liver ammonia was higher in animals supplemented with DIP compared with the SED group (*p* = 0.008) (Figure 5).

### 3.6. Maximum Carrying Capacity (MCC) Tests

**Figure 6:**
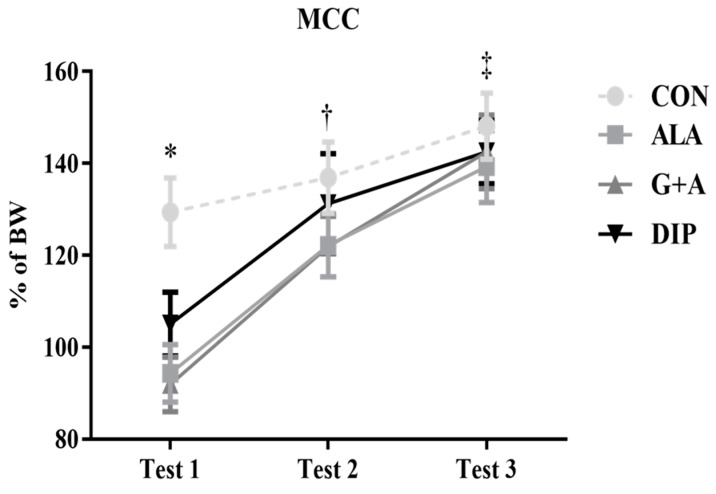
Maximum carrying capacity tests of rats submitted to resistance exercise and supplementation with l-glutamine and l-alanine. SED: sedentary group, CON: trained group, ALA: trained and supplemented with alanine, G + A: trained and supplemented with glutamine and alanine in their free form, and DIP: trained and supplemented with l-alanyl-l-glutamine. * *p* < 0.05 vs. ALA and G + A. † *p* < 0.05 vs. test 1. ‡ *p* < 0.05 vs. test 1 and 2. Data are presented as mean ± SD.

In the first MCC test, the CON group presented a higher weightlifting capacity than the ALA and G + A groups (*p* = 0.005); even so, there was no difference between the groups in the subsequent tests. The performance of all groups was improved in the second and third tests, compared to the first one (*p* < 0.001). The amino acid supplementation in free or conjugated form did not affect exercise performance (Figure 6).

## 4. Discussion

The current study investigated the effects of glutamine and alanine supplementation on central fatigue parameters in rats submitted to RT. To the best of our knowledge, this is the first study that has evaluated these amino acids using the central fatigue hypothesis. BCAA and carbohydrate administration are common nutritional strategies used in an attempt to alleviate central fatigue, though these interventions present certain deleterious effects, such as hyperammonemia (BCAA supplementation) and increased insulin release, augmenting the muscle uptake of BCAA (carbohydrate supplementation) [6,13,18,42,43,44,45,46].

In our study, glutamine and alanine administration did not affect food consumption and body weight gain, allowing us to evaluate the exclusive effect of these amino acids on trained animals. Although supplemented animals ingested more fluid, there was no statistically significant difference between groups in the plasma concentrations of glutamine and alanine. Similar to our findings, Rogero et al., [33] observed that chronic oral supplementation with free glutamine and l-alanyl-l-glutamine did not affect glutaminemia. Probably, increased plasma glutamine and alanine concentrations after supplementation stimulate the tissue uptake of these amino acids, increasing their stores and maintaining plasma concentrations at basal values [33].

RT increased glycemia and reduced liver glycogen levels, but, on the other hand, supplementation did not affect glycemia and increased muscle glycogen content. Several metabolic processes such as gluconeogenesis and glycogenolysis, are optimized during exercise to provide glucose to cells and tissues, increasing glycemia and reducing glycogen content [20,23]. Decreases in energy substrates and stores of glucose for adenosine triphosphate (ATP) production are important causes of fatigue [2,47], and evidence indicates that the reduction of glucogenesis is directly associated with exercise performance impairment [47]. Glutamine and alanine are considered the most glycogenic amino acids in humans and animals [20,22,48]. Bowtell and Bruce [47] observed that glutamine supplementation increased muscle concentrations of Krebs cycle precursors such as citrate, malate, fumarate, and succinate after ten minutes of cycling. Moreover, glutamine is a direct stimulator of glycogen synthesis via the activation of glycogen synthase and the diversion of carbon from glutamine to glycogen [20], and its supplementation was seen to restore muscle glycogen at the same level as glucose supplementation in a glycogen-depleting exercise protocol [47].

These amino acids are also associated with the prevention of hyperammonemia through physical exercise [23]. As described herein, RT increased muscle and liver ammonia, whereas G + A prevented the increase in muscle ammonia through RT, and DIP supplementation was the main factor in increasing ammonia in the liver. Ammonia is considered an important fatigue marker since this metabolite is toxic and can lead to deleterious alterations in the brain, such as the impairment of cerebral energy metabolism and neurotransmission, affecting motor control [4,8]. Thus, the prevention of hyperammonemia could avoid central nervous system (CNS) injuries and increase exercise performance [23].

Ammonia production in physical exercise occurs via amino acid oxidation and during energy metabolism (adenosine monophosphate—AMP deamination), indicating the reduction of ATP concentration and glycogen content [2,4]. Reduced glycogen stores increase the use of amino acids as an energy source. Since amino acid oxidation increases ammonia synthesis [4,23], it could be inferred that DIP was used as an important hepatic energy substrate due to glycogen depletion. It is worth mentioning that, despite increasing liver ammonia, DIP supplementation did not promote hyperammonemia. Glutamine and alanine are considered the main transporters of nitrogen (ammonia) in the body, preventing the muscle accumulation of this metabolite, and favoring ammonia hepatic metabolism, as well as its renal excretion [25,49]. Glutamine can also reduce ammonia indirectly by arginine synthesis [50] as arginine promotes vasodilation and increases urea cycle activity, directing accumulated ammonia to be metabolized [18,19,51].

Although energy availability reduction and ammonia accumulation are important causes of fatigue, the central fatigue hypothesis is based on parameters linked to the brain synthesis of serotonin. As previously mentioned, RT and supplementation with l-alanyl-l-glutamine (DIP), but not with free amino acids (ALA and G + A), increased plasma FFA content. Reduction of energy stores (especially glycogen) during exercise increases FFA mobilization disproportionally, over the rate of muscle transportation, markedly increasing plasma FFA [8,11,14]. This explains the effect of RT on reducing hepatic glycogen content and increasing plasma FFA.

ALA and G + A administration decreased plasma FFA concentrations. Glutamine and alanine present an important lipogenic role as white adipose tissue, using glutamine in high amounts for glutamate synthesis, which is converted to acetyl-CoA, allowing lipid synthesis (lipogenesis) [52]. FFA produced by glutamine is incorporated into triacylglycerol in adypocytes. Aside from being an important substrate for lipogenesis, glutamine also regulates the activity of FFA synthetase (FAS) and glycerophosphate dehydrogenase (GPDH) through glutamine metabolism products, such as glucosamine-6-phosphate [22]. Likewise, alanine is a precursor of acetyl-CoA [47]; thus, it is possible that this amino acid also contributes to lipogenesis [52]. Therefore, similarly to carbohydrate supplementation [53,54], these glucogenic amino acids in their free form reduced lipolysis and, hence, plasma FFA.

Surprisingly, DIP supplementation increased FFA plasma levels. Some results obtained in the present study differed according to the supplementation form (amino acids in their free form or conjugated). This may be related to the fact that dipeptides (such as l-alanyl-l-glutamine) are absorbed by the luminal membrane in their intact form by the oligopeptide transporter PepT-1. Thus, the dipeptide form (l-alanyl-l-glutamine) may be present in blood and certain tissues, playing different roles compared with free amino acids [33,55]. Therefore, additional studies need to be conducted to further elucidate the effect of l-alanyl-l-glutamine on lipolysis rates during physical exercise.

The increase in FFA concentrations displaces tryptophan from albumin, increasing free tryptophan [8,12,13,14,15,16,17]. Thus, DIP group presented the highest levels of plasma FFA and the free tryptophan/total tryptophan ratio, indicating a large proportion of free tryptophan in this group. In addition, there was an increase in hypothalamic serotonin content in the CON and DIP groups. Increased brain serotonin concentrations are linked to several behavioral alterations, such as anxiety, aggression, reduced appetite, sleepiness, and fatigue, reducing the mental and physical efficiency of athletes [11,53,56,57]. Several studies confirm that increased serotonin synthesis impairs exercise performance [9,11,58,59] but others failed to demonstrate this relation [13,43,57,60,61,62,63].

Herein, the exercise performance was evaluated by MCC tests. In the first test, the CON group presented a higher weightlifting capacity compared with ALA and G + A groups. This result is surprising since animals had similar baseline characteristics, such as body weight (Table 1). The first MCC test was very stressful due to the high load increase (5% of BW in the adaptation to 75% of BW in the MCC), and, as the rats performed the test in the same room, it is possible that the first animals were less stressed and had a better performance than the last ones. Despite this result, there was no statistically significant difference between groups in the subsequent MCC tests (2 and 3), indicating the adaptation of the animals and the similarity between groups in regard to muscle strength.

In our study, increased serotonin levels were not markedly associated to physical performance. Even though the animals from the DIP group had the highest concentration of plasma FFA, free tryptophan/total tryptophan ratio and serotonin in the hypothalamus, there was no difference in the MCC tests between groups. These findings may indicate that brain serotonin concentration is not crucial for promoting fatigue. However, it is worth noting that the increase in serotonin synthesis is not necessarily associated with increasing the serotonin release. Thus, the increased brain serotonin concentration may not affect neurotransmission and, consequently, physical performance [57,64]. Moreover, brain function is not determined by a single neurotransmitter, and the interaction between brain serotonin and dopamine has also been explored as having a regulative role in the development of exercise fatigue. Thus, the serotonin/dopamine ratio is considered a more accurate parameter of central fatigue [8,9].

Dopamine is a neurotransmitter that plays an important role in motivation, memory, rewards, attention, and participates in motor control [57,65], presenting an interesting function in improving performance [66,67,68,69,70,71]. Briefly, serotonin and dopamine play almost opposite biological functions [8]. Dopamine brain concentrations tend to remain stable in stress situations in rodents [72], explaining the absence of difference between groups at hypothalamic dopamine levels. Furthermore, the dopamine precursor, tyrosine [56,68], did not change with RT or glutamine and alanine administration. Consequently, similar to our findings of hypothalamic serotonin, the ratio serotonin/dopamine in hypothalamus was higher in CON and DIP groups.

## 5. Conclusions

The main findings of the present study are: (i) RT increased the concentrations of plasma FFA and hypothalamic serotonin; (ii) the increase in brain serotonin content and the brain serotonin/dopamine ratio are not crucial for impairing exercise performance in RT; and (iii) glutamine and alanine supplementation could improve (ALA and G + A) or impair (DIP) central fatigue parameters depending on their supplementation form.

## Figures and Tables

**Table 1:** Food consumption, fluid intake and body weight gain.

Parameter	SED	CON	ALA	G + A	DIP	*p*
Food consumption (g/week)	189.5 ± 18.0	173.0 ± 7.9	170.6 ± 12.6	173.2 ± 11.3	170.1 ± 9.8	0.082
Fluid intake (mL/week)	270.2 ± 28.1	295.2 ± 25.7	326.4 ± 23.5	355.8 ± 25.7 *	310.2 ± 14.6	<0.001
Baseline body weight (g)	325.5 ± 19.9	298.8 ± 38.4	290.7 ± 25.7	302.6 ± 19.6	290.8 ± 17.3	0.056
Body weight gain (g/week)	16.5 ± 4.1	11.9 ± 3.5 ^†^	14.9 ± 2.5	14.7 ± 1.9	14.5 ± 2.8	0.044

SED: sedentary group, CON: trained group, ALA: trained and supplemented with alanine, G + A: trained and supplemented with glutamine and alanine in their free form, and DIP: trained and supplemented with l-alanyl-l-glutamine. * *p* < 0.05 vs. SED and CON. † *p* < 0.05 vs. SED. Data are presented as mean ± SD.

**Table 2:** Plasma concentrations in amino acids.

Amino Acids (mmol/L)	SED	CON	ALA	G + A	DIP	*p*
Alanine	0.81 ± 0.15	0.75 ± 0.09	0.79 ± 0.11	0.82 ± 0.09	0.82 ± 0.13	0.724
Arginine	0.68 ± 0.08	0.67 ± 0.11	0.62 ± 0.08	0.62 ± 0.06	0.63 ± 0.05	0.468
Asparagine	0.21 ± 0.02	0.19 ± 0.02	0.19 ± 0.03	0.19 ± 0.02	0.19 ± 0.03	0.590
Aspartate	0.17 ± 0.04	0.13 ± 0.02	0.14 ± 0.02	0.15 ± 0.02	0.15 ± 0.03	0.214
Cysteine	0.04 ± 0.01	0.03 ± 0.01	0.03 ± 0.01	0.04 ± 0.01	0.04 ± 0.01	0.718
Glutamate	1.60 ± 0.21	1.44 ± 0.18	1.47 ± 0.22	1.61 ± 0.14	1.68 ± 016	0.073
Glutamine	0.84 ± 0.28	1.04 ± 0.26	0.98 ± 0.16	0.85 ± 0.22	0.88 ± 0.19	0.327
Glycine	0.52 ± 0.07	0.47 ± 0.04	0.48 ± 0.11	0.48 ± 0.04	0.53 ± 0.04	0.346
Histidine	0.23 ± 0.02	0.23 ± 0.02	0.21 ± 0.03	0.20 ± 0.02	0.20 ± 0.02	0.051
Isoleucine	0.30 ± 0.05	0.27 ± 0.04	0.28 ± 0.05	0.24 ± 0.04	0.26 ± 0.03	0.097
Leucine	0.55 ± 0.09	0.49 ± 0.06	0.54 ± 0.08	0.47 ± 0.06	0.50 ± 0.07	0.273
Lysine	1.15 ± 0.09	1.11 ± 0.12	1.04 ± 0.08	1.14 ± 0.12	1.07 ± 0.11	0.205
Methionine	0.19 ± 0.02	0.19 ± 0.02	0.20 ± 0.02	0.20 ± 0.02	0.19 ± 0.01	0.976
Phenylalanine	0.30 ± 0.03	0.29 ± 0.03	0.30 ± 0.01	0.28 ± 0.03	0.26 ± 0.03	0.089
Proline	0.48 ± 0.04	0.43 ± 0.04	0.48 ± 0.09	0.48 ± 0.06	0.44 ± 0.02	0.165
Serine	0.49 ± 0.06	0.46 ± 0.05	0.46 ± 0.05	0.45 ± 0.04	0.48 ± 0.07	0.603
Taurine	0.83 ± 0.11	0.77 ± 0.13	0.73 ± 0.13	0.79 ± 0.10	0.83 ± 0.06	0.452
Threonine	0.42 ± 0.09	0.37 ± 0.05	0.38 ± 0.08	0.36 ± 0.08	0.34 ± 0.05	0.250
Tyrosine	0.29 ± 0.06	0.26 ± 0.05	0.27 ± 0.06	0.27 ± 0.05	0.26 ± 0.04	0.938
Valine	0.62 ± 0.08	0.55 ± 0.04	0.57 ± 0.06	0.53 ± 0.05	0.54 ± 0.07	0.078
Total tryptophan	0.009 ± 0.002	0.010 ± 0.003	0.013 ± 0.005	0.007 ± 0.005	0.006 ± 0.005 *	0.022
Free tryptophan	0.003 ± 0.002	0.005 ± 0.003	0.002 ± 0.001	0.003 ± 0.001	0.004 ± 0.002	0.097
Free tryptophan/total tryptophan ratio	0.51 ± 0.32	0.65 ± 0.14	0.32 ± 0.22	0.30 ± 0.14	1.64 ± 1.38 ^†^	0.017
Free tryptophan/BCAA ratio	0.001 ± 0.001	0.003 ± 0.002	0.001 ± 0.001	0.002 ± 0.001	0.003 ± 0.002	0.147

SED: sedentary group, CON: trained group, ALA: trained and supplemented with alanine, G + A: trained and supplemented with glutamine and alanine in their free form, and DIP: trained and supplemented with l-alanyl-l-glutamine. * *p* < 0.05 vs. ALA. † *p* < 0.05 vs. ALA and G + A. Data are presented as mean ± SD. BCAA: branched-chain amino acids.

**Table 3:** Glycemia before and after training.

Glycemia (mg/dL)	CON	ALA	G + A	DIP	*p*
Pre-training 25% of BW	112.0 ± 11.68	111.9 ± 6.52	118.1 ± 9.00	113.0 ± 18.86	0.715
Post-training 25% of BW	120.4 ± 15.84	122.9 ± 9.52	130.0 ± 14.97	128.8 ± 13.52	0.473
Pre-training 50% of BW	83.0 ± 5.68	80.4 ± 3.19	85.8 ± 4.35	85.9 ± 5.06	0.097
Post-training 50% of BW	97.7 ± 9.26 *	94.7 ± 4.51 *	99.8 ± 14.05 *	97.5 ± 7.87 *	0.791
Pre-training 75% of BW	95.8 ± 5.58	93.3 ± 12.94	97.5 ± 5.91	94.1 ± 5.14	0.729
Post-training 75% of BW	115.6 ± 16.24 *	106.6 ± 20.34 *	118.2 ± 11.44 *	118.2 ± 8.83 *	0.416
Pre-training 100% of BW	89.2 ± 11.70	89.7 ± 2.67	97.7 ± 4.51	91.0 ± 8.07	0.132
Post-training 100% of BW	109.9 ± 12.70 *	109.4 ± 9.97 *	117.3 ± 12.99 *	115.7 ± 9.92 *	0.448
One hour after the last training	146.4 ± 25.13	145.5 ± 13.11	152.7 ± 18.43	155.6 ± 16.55	0.791

CON: trained group, ALA: trained and supplemented with alanine, G + A: trained and supplemented with glutamine and alanine in their free form, and DIP: trained and supplemented with l-alanyl-l-glutamine. * Significant difference between times (before and after training) in all groups (*p* < 0.05). Data are presented as mean ± SD. BW: Body weight.

## References

[1] Béquet F., Gomez-Merino D., Berthelot M., Guezennec C.Y. (2002). Evidence that brain glucose availability influences exercise-enhanced extracellular 5-HT level in hippocampus: A microdialysis study in exercising rats. Acta Physiol. Scand..

[2] Finsterer J. (2012). Biomarkers of peripheral muscle fatigue during exercise. BMC Musculoskelet. Disord..

[3] Newsholme E.A., Blomstrand E. (2006). Branched-Chain Amino Acids and Central Fatigue. J. Nutr..

[4] Wilkinson D.J., Smeeton N.J., Watt P.W. (2010). Ammonia metabolism, the brain and fatigue; Revisiting the link. Prog. Neurobiol..

[5] Chaouloff F., Laude D., Guezennec Y., Elghozi J.L. (1986). Motor Activity Increases Tryptophan, 5-Hydroxyindoleacetic Acid, and Homovanillic Acid in Ventricular Cerebrospinal Fluid of the Conscious Rat. J. Neurochem..

[6] Blomstrand E., Celsing F., Newsholme E.A. (1988). Changes in plasma concentrations of aromatic and branched—Chain amino acids during sustained exercise in man and their possible role in fatigue. Acta Physiol. Scand..

[7] Smriga M., Kameishi M., Tanaka T., Kondoh T., Torii K. (2002). Preference for a solution of branched-chain amino acids plus glutamine and arginine correlates with free running activity in rats: Involvement of serotonergic-dependent processes of lateral hypothalamus. Nutr. Neurosci..

[8] Meeusen R., Watson P., Hasegawa H., Roelands B., Piacentini M.F. (2006). Central fatigue: The serotonin hypothesis and beyond. Sports Med..

[9] Cordeiro L.M.S., Guimarães J.B., Wanner S.P., La Guardia R.B., Miranda R.M., Marubayashi U., Soares D.D. (2014). Inhibition of tryptophan hydroxylase abolishes fatigue induced by central tryptophan in exercising rats. Scand. J. Med. Sci. Sports.

[10] Blomstrand E., Perrett D., Parry-Billings M., Newsholme E.A. (1989). Effect of sustained exercise on plasma amino acid concentrations and on 5-hydroxytryptamine metabolism in six different brain regions in the rat. Acta Physiol. Scand..

[11] Weicker H., Struder H.K. (2001). Influence of exercis on serotonergic neuromeodulation in the brain. J. Amin. Acids.

[12] Skeie B., Kvetan V., Gil K.M., Rothkopf M.M., Newsholme E.A., Askanazi J. (1990). Branch-chain amino acids: Their metabolism and clinical utility. Crit. Care Med..

[13] Verger P., Aymard P., Cynobert L., Anton G., Luigi R. (1994). Effects of administration of branched-chain amino acids vs. glucose during acute exercise in the rat. Physiol. Behav..

[14] Blomstrand E. (2001). Amino acids and central fatigue. Amino Acids.

[15] Blomstrand E., Møller K., Secher N.H., Nybo L. (2005). Effect of carbohydrate ingestion on brain exchange of amino acids during sustained exercise in human subjects. Acta Physiol. Scand..

[16] Fernstrom J.D. (2005). Branched-Chain Amino Acids and Brain Function. Proceedings of the 4th Amino Acid Assessment Workshop.

[17] Blomstrand E., Hassmén P., Ek S., Ekblom B., Newsholme E.A. (1997). Influence of ingesting a solution of branched-chain amino acids on perceveid exertion during exercise. Acta Physiol. Scand..

[18] Chang C.K., Chien K.M.C., Chang J.H., Huang M.H., Liang Y.C., Liu T.H. (2015). Branched-chain amino acids and arginine improve performance in two consecutive days of simulated handball games in male and female athletes: A randomized trial. PLoS ONE.

[19] Chen I.-F., Wu H.-J., Chen C.-Y., Chou K.-M., Chang C.-K. (2016). Branched-chain amino acids, arginine, citrulline alleviate central fatigue after 3 simulated matches in taekwondo athletes: A randomized controlled trial. J. Int. Soc. Sports Nutr..

[20] Stumvoll M., Perriello G., Meyer C., Gerich J. (1999). Role of glutamine in human carbohydrate metabolism in kidney and other tissues. Kidney Int..

[21] Rennie M.J., Bowtell J.L., Bruce M., Khogali S.E.O. (2001). Glutamine Metabolism: Nutritional and Clinical Significance Interaction between Glutamine Availability and Metabolism of Glycogen, Tricarboxylic Acid Cycle Intermediates and Glutathione 1, 2. J. Nutr..

[22] Curi R., Lagranha C.J., Doi S.Q., Sellitti D.F., Procopio J., Pithon-Curi T.C., Corless M., Newsholme P. (2005). Molecular mechanisms of glutamine action. J. Cell. Physiol..

[23] Bassini-Cameron A., Monteiro A., Gomes A., Werneck-de-Castro J.P.S., Cameron L. (2008). Glutamine protects against increases in blood ammonia in football players in an exercise intensity-dependent way. Br. J. Sports Med..

[24] Roth E., Oehler R., Manhart N., Exner R., Wessner B., Strasser E., Spittler A. (2002). Regulative potential of glutamine—Relation to glutathione metabolism. Nutrition.

[25] Newsholme P., Procopio J., Ramos Lima M.M., Pithon-Curi T.C., Curi R. (2003). Glutamine and glutamate—Their central role in cell metabolism and function. Cell Biochem. Funct..

[26] Guezennec C.Y., Abdelmalki A., Merino D., Bigord X., Berthelot M., Pierard C., Peres M. (1998). Effects of prolonged exercise on brain ammonia and amino acids. Int. J. Sports Med..

[27] Raizel R., Leite J.S.M., Hypólito T.M., Coqueiro A.Y., Newsholme P., Cruzat V.F., Tirapegui J. (2016). Determination of the anti-inflammatory and cytoprotective effects of l-glutamine and l-alanine, or dipeptide, supplementation in rats submitted to resistance exercise. Br. J. Nutr..

[28] Leite J.S.M., Raizel R., Hypólito T.M., Rosa T.D.S., Cruzat V.F., Tirapegui J. (2016). l-glutamine and l-alanine supplementation increase glutamine-glutathione axis and muscle HSP-27 in rats trained using a progressive high-intensity resistance exercise. Appl. Physiol. Nutr. Metab..

[29] Coqueiro A.Y., Raizel R., Hypólito T.M., Tirapegui J. (2017). Effects of supplementation with l-glutamine and L-alanine in the body composition of rats submitted to resistance exercise. Rev. Bras. Cienc. Esporte.

[30] Curi T.C., De Melo M.P., De Azevedo R.B., Zorn T.M., Curi R. (1997). Glutamine utilization by rat neutrophils: Presence of phosphate-dependent glutaminase. Am. J. Physiol..

[31] Rogero M.M., Tirapegui J., Pedrosa R.G., de Castro I.A., de Oliveira Pires I.S. (2006). Effect of alanyl-glutamine supplementation on plasma and tissue glutamine concentrations in rats submitted to exhaustive exercise. Nutrition.

[32] Gleeson M. (2008). Dosing and efficacy of glutamine supplementation in human exercise and sport training. J. Nutr..

[33] Rogero M.M., Tirapegui J., Pedrosa R.G., De Oliveira Pires I.S., De Castro I.A. (2004). Plasma and tissue glutamine response to acute and chronic supplementation with l-glutamine and l-alanyl-l-glutamine in rats. Nutr. Res..

[34] Cruzat V.F., Tirapegui J. (2009). Effects of oral supplementation with glutamine and alanyl-glutamine on glutamine, glutamate, and glutathione status in trained rats and subjected to long-duration exercise. Nutrition.

[35] Scheffer D.L., Silva L.A., Tromm C.B., da Rosa G.L., Silveira P.C.L., de Souza C.T., Latini A., Pinho R.A. (2012). Impact of different resistance training protocols on muscular oxidative stress parameters. Appl. Physiol. Nutr. Metab..

[36] Hornberger T.A., Farrar R.P. (2004). Physiological Hypertrophy of the FHL Muscle Following 8 Weeks of Progressive Resistance Exercise in the Rat. Can. J. Appl. Physiol..

[37] Sanches I.C., Conti F.F., Sartori M., Irigoyen M.C., De Angelis K. (2014). Standardization of resistance exercise training: Effects in diabetic ovariectomized rats. Int. J. Sports Med..

[38] Petry É.R., Cruzat V.F., Heck T.G., Leite J.S.M., De Bittencourt P.I.H., Tirapegui J. (2014). Alanyl-glutamine and glutamine plus alanine supplements improve skeletal redox status in trained rats: Involvement of heat shock protein pathways. Life Sci..

[39] Cruzat V.F., Rogero M.M., Tirapegui J. (2010). Effects of supplementation with free glutamine and the dipeptide alanyl-glutamine on parameters of muscle damage and inflammation in rats submitted to prolonged exercise. Cell Biochem. Funct..

[40] Prada P.O., Hirabara S.M., De Souza C.T., Schenka A.A., Zecchin H.G., Vassallo J., Velloso L.A., Carneiro E., Carvalheira J.B.C., Curi R. (2007). l-glutamine supplementation induces insulin resistance in adipose tissue and improves insulin signalling in liver and muscle of rats with diet-induced obesity. Diabetologia.

[41] Hassid W., Albrahams S. (1957). Chemical procedures for analyses of polisacchardies methods enzimol. Methods Enzim..

[42] MacLean D.A., Graham T.E., Saltin B. (1996). Stimulation of muscle ammonia production during exercise following branched-chain amino acid supplementation in humans. J. Physiol..

[43] Strüder H., Hollmann W., Platen P., Donike M., Gotzmann A., Weber K. (1998). Influence of Paroxetine, Branched-Chain Amino Acids and Tyrosine on Neuroendocrine System Responses and Fatigue in Humans. Horm. Metab. Res..

[44] Davis J.M., Welsh R.S., De Volve K.L., Alderson N.A. (1999). Effects of branched-chain amino acids and carbohydrate on fatigue during intermittent, high-intensity running. Int. J. Sports Med..

[45] Watson P., Shirreffs S.M., Maughan R.J. (2004). The effect of acute branched-chain amino acid supplementation on prolonged exercise capacity in a warm environment. Eur. J. Appl. Physiol..

[46] Kim D.-H., Kim S.-H., Jeong W.-S., Lee H.-Y. (2013). Effect of BCAA intake during endurance exercises on fatigue substances, muscle damage substances, and energy metabolism substances. J. Exerc. Nutr. Biochem..

[47] Bowtell J.L., Bruce M. (2002). Glutamine: An Anaplerotic Precursor. Nutrition.

[48] Iwashita S., Williams P., Jabbour K., Ueda T., Kobayashi H., Baier S., Flakoll P.J. (2005). Impact of glutamine supplementation on glucose homeostasis during and after exercise. J. Appl. Physiol..

[49] Bode B.P. (2001). Recent Molecular Advances in Mammalian Glutamine Transport. J. Nutr..

[50] Melis G.C., ter Wengel N., Boelens P.G., van Leeuwen P.A.M. (2004). Glutamine: Recent developments in research on the clinical significance of glutamine. Curr. Opin. Clin. Nutr. Metab. Care.

[51] Schaefer A., Piquard F., Geny B., Doutreleau S., Lampert E., Mettauer B., Lonsdorfer J. (2002). L-arginine reduces exercise-induced increase in plasma lactate and ammonia. Int. J. Sports Med..

[52] Kowalchuk J.M., Curi R., Newsholme E.A. (1988). Glutamine metabolism in isolated incubated adipocytes of the rat. Biochem. J..

[53] Davis J.M., Bailey S.P., Woods J.A., Galiano F.J., Hamilton M.T., Bartoli W.P. (1992). Applied Physiology on plasma free tryptophan and branched, chain amino acids during prolonged cycling. Eur. J. Appl. Physiol..

[54] Jang T.-R., Wu C.-L., Chang C.-M., Hung W., Fang S.-H., Chang C.-K. (2011). Effects of carbohydrate, branched-chain amino acids, and arginine in recovery period on the subsequent performance in wrestlers. J. Int. Soc. Sports Nutr..

[55] Thamotharan M., Bawani S.Z., Zhou X., Adibi S.A. (1999). Functional and molecular expression of intestinal oligopeptide transporter (Pept-1) after a brief fast. Metabolism.

[56] Fernstrom J.D. (1994). Dietary amino acids and brain function. J. Am. Diet. Assoc..

[57] Meeusen R., Thorré K., Chaouloff F., Sarre S., De Meirleir K., Ebinger G., Michotte Y. (1996). Effects of tryptophan and/or acute running on extracellular 5-HT and 5-HIAA levels in the hippocampus of food-deprived rats. Brain Res..

[58] Bailey S.P., Davis J.M., Ahlborn E.N. (1993). Endurance Performance in the Rat. Int. J. Sports Med..

[59] Marvin G., Sharma A., Aston W., Field C., Kendall M.J., Jones D.A. (1997). The effects of buspirone on perceived exertion and time to fatigue in man. Exp. Physiol..

[60] Van Hall G., Raaymakers J.S.H., Saris W.H.M., Wagenmakers A.J.M. (1995). Ingestion of branched-chain amino acids and tryptophan during sustained exercise in man: failure to affect performance. J. Physiol..

[61] Pannier J.L., Bouckaert J.J., Lefebvre R.A. (1995). The antiserotonin agent pizotifen does not increase endurance performance in humans. Eur. J. Appl. Physiol. Occup. Physiol..

[62] Piacentini M.F., Meeusen R., Buyse L., de Schutter G., de Meirleir K. (2002). No effect of a selective serotonergic/noradrenergic reuptake inhibitor on endurance performance. Eur. J. Sport Sci..

[63] Hobson R.M., Watson P., Maughan R.J. (2013). Acute tryptophan depletion does not improve endurance cycling capacity in a warm environment. Amino Acids.

[64] Cunliffe A., Obeid O.A., Powell-Tuck J. (1998). A placebo controlled investigation of the effects of tryptophan or placebo on subjective and objective measures of fatigue. Eur. J. Clin. Nutr..

[65] Piacentini M.F. (2004). Hormonal responses during prolonged exercise are influenced by a selective DA/NA reuptake inhibitor. Br. J. Sports Med..

[66] Gerald M.C. (1978). Effects of (+)-amphetamine on the treadmill endurance performance of rats. Neuropharmacology.

[67] Heyes M.P., Garnett E.S., Coates G. (1985). Central dopaminergic activity influences rats ability to exercise. Life Sci..

[68] Hattori S., Naoi M., Nishino H. (1994). Striatal dopamine turnover during treadmill running in the rat: Releation to the speed of running. Brain Res. Bull..

[69] Watson P., Hasegawa H., Roelands B., Piacentini M.F., Looverie R., Meeusen R. (2005). Acute dopamine/noradrenaline reuptake inhibition enhances human exercise performance in warm, but not temperate conditions. J. Physiol..

[70] Roelands B., Hasegawa H., Watson P., Piacentini M.F., Buyse L., De Schutter G., Meeusen R.R. (2008). The effects of acute dopamine reuptake inhibition on performance. Med. Sci. Sports Exerc..

[71] Meeusen R., Roelands B. (2010). Central fatigue and neurotransmitters, can thermoregulation be manipulated?. Scand. J. Med. Sci. Sports.

[72] Bliss E.L., Ailion J. (1971). Relationship of stress and activity to brain dopamine and homovanillic acid. Life Sci..

